# In vivo confocal microscopic and histological findings of unknown bullous keratopathy probably associated with pseudoexfoliation syndrome

**DOI:** 10.1186/1471-2415-12-17

**Published:** 2012-06-22

**Authors:** Xiaodong Zheng, Yasushi Inoue, Atsushi Shiraishi, Yoko Hara, Tomoko Goto, Yuichi Ohashi

**Affiliations:** 1 Department of Ophthalmology, Ehime University School of Medicine, Toon city, Ehime, 791-0295, Japan; 2 Inoue Eye Clinic, Uno 1-14-31, Tamano City, Okayama, Japan; 3 Department of Ophthalmology, Takanoko Hospital, Matsuyama, Ehime, Japan

**Keywords:** Cornea, Bullous keratopathy, Pseudoexfoliation syndrome, Electron microscopy, In vivo confocal microscopy

## Abstract

**Background:**

Bullous keratopathy (BK), a severe sight-threatening disorder can have a variety of etiologies such as prophylactic laser iridotomy, intraocular surgery, trauma, and other ocular disorders. However, there are cases of unknown origins, among which a unique clinical entity namely pseudoexfoliation syndrome (PEX) is having increased importance.

**Case presentation:**

In this case note, we report the clinical features and in vivo confocal microscopic and pathological findings of two BK cases of unknown cause.

**Conclusions:**

Our findings suggest that the BK was caused by the corneal endotheliopathy of PEX, a common disease that could affect up to 30% of people over 60 years old and is more prevalent than we have believed.

## Background

Bullous keratopathy (BK), a sight-threatening disorder caused by endothelial cell dysfunction, is one of the leading causes for corneal transplantation in many countries. A recent national survey in Japan showed the most common cause of BK was cataract surgery, followed by prophylactic laser iridotomy, glaucoma surgery, trauma, and other ocular diseases [1]. However, there are cases of which the etiology is unknown. In this note, we report the clinical features and in vivo confocal microscopic (IVCM) and pathological findings of two BK patients diagnosed with an unknown cause. Our findings suggest that the BK was probably caused by the corneal endotheliopathy of pseudoexfoliation syndrome (PEX), a common disease that could affect up to 30% of people over 60 years old [2].

## Case presentation

### Case 1

An 82-year-old man complained of blurred vision in his right eye of one year duration visited our hospital. He had no history of intraocular surgery and trauma, and his medical records showed that he was not taking any medication, such as major tranquilizers, that could cause corneal endotheliopathy.

**Figure 1:**
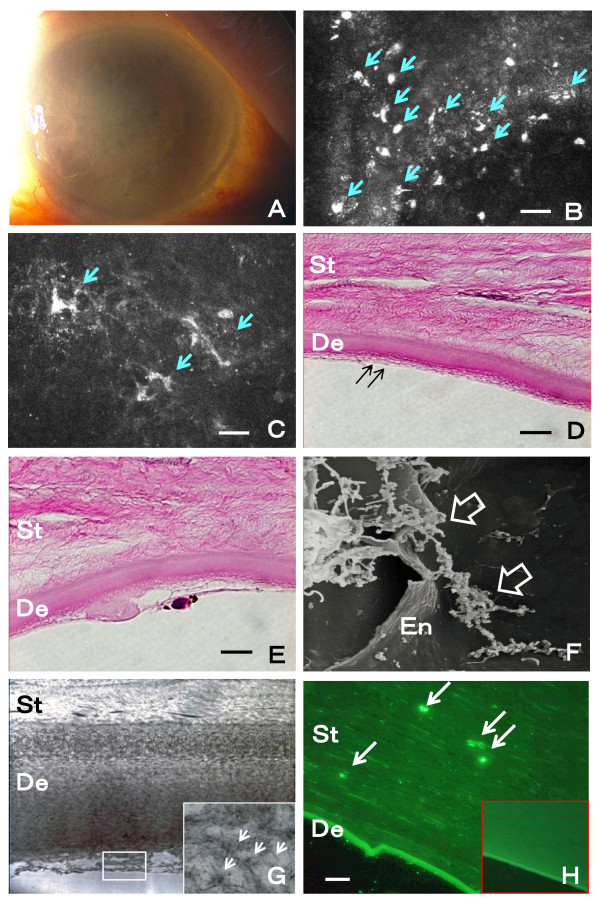
**Findings in a patient with unknown bullous keratopathy (Case 1).** A. Slit-lamp photograph showing diffused corneal edema. B and C. In vivo confocal microscopy of the endothelial layer showing abundant irregular hyperreflective materials most likely PEX materials in the endothelium (B, arrows; Bar = 50 μm) and stroma of the cornea (C, arrows; Bar = 50 μm). D and E. Hematoxylin-eosin staining of light microscopy showing apparent loss of endothelium and formation of fibrillar layer (D, double arrows) and the fibroblast-like change of an endothelium (E). St: stroma, De: Descemet membrane, Bar = 10 μm. F and G. Scanning electronic microscopy showing PEX fibers (open arrows) adjacent to destroyed endothelium (F; En: endothelium) and transmission electron microscopic identification of PEX fibers (arrows in enlarge window) in the thickened Descemet membrane (G; St: stroma, De: Descemet membrane, original magnification: x5000). H. Anti-LOXL1 immunohistochemical staining showing positive staining within the stroma of the cornea (arrows). St: stroma, De: Descemet membrane, Bar = 10 μm. Window image shows negative control staining without LOXL1 antibody.

On our examination, his best-corrected visual acuity (BCVA) was 0.04 (Snellen: 20/500) OD and 0.8 (20/25) OS. The intraocular pressure was 12 mmHg OD and 10 mmHg OS. The right cornea was diffusely edematous and the details of the iris and lens were barely visible (Figure 1A). No inflammatory signs were found in the anterior chamber and vitreous, and the retinal reflex was normal. IVCM showed characteristic changes of the endothelial cells with increased polymorphism and pleomegatism. Abundant PEX materials were found deposited on the corneal endothelial cells (Figure 1B). It was noteworthy that PEX materials were also found in the subbasal layer and stroma of the cornea (Figure 1C).

With an informed consent, this patient underwent penetrating keratoplasty in combination with cataract extraction and intraocular lens insertion. He recovered well with a clear corneal graft and his final BCVA was 0.8 (Snellen 20/25) at his final visit to our department at 3 years after surgery.

The removed cornea was processed for pathology. Histological study showed that there was a marked loss of endothelial cells, formation of fibrillar layer (Figure 1D), and fibroblast-like changes of the remaining cells (Figure 1E). Electron microscopy identified abundant PEX materials deposited on the corneal endothelium and within the thickened Descemet layer (Figures 1F and 1G). Immunohistochemical studies using anti-LOXL1 antibody confirmed positive staining correlating with the PEX materials deposited in the stroma (Figure 1H).

### Case 2

**Figure 2:**
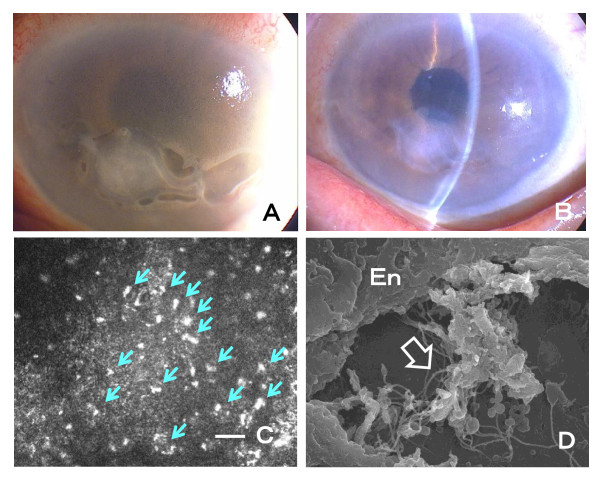
**Findings in another patient with unknown bullous keratopathy (Case 2).** A. Slit-lamp photograph showing diffused corneal edema and giant epithelial bullae. B. Slit-lamp photograph showing PEX materials on the pupillary iris. C. In vivo confocal microscopy showing PEX materials on the endothelial cells (arrows; Bar = 50 μm). D. Scanning electronic microscopy showing fabric PEX materials (open arrow) adjacent to a destroyed endothelium. En: endothelium, original magnification: x5000.

A 76-year-old woman had decreased vision in her left eye and was diagnosed with BK of unknown cause. She also had no history of intraocular surgery, laser therapy, and trauma before the onset of BK. On her visit to our hospital, her BCVA was 0.1 (Snellen 20/200) in the left eye, and the intraocular pressure was 13 mmHg. Diffused corneal edema was present and there were no signs of inflammation in this eye (Figure 2A). Both of her eyes had PEX materials in the pupillary area and right lens capsule (Figure 2B). IVCM showed typical PEX materials deposits on the endothelial layers and the anterior stroma of the edematous cornea (Figure 2C).

DSAEK was performed and the removed endothelial layer was examined by electron microscopy. Extensive PEX materials were found on the destroyed endothelial cell layer (Figure 2D). She recovered uneventful after the surgery and her BCVA was 0.9 (Snellen 20/22) at her latest visit 9 month after the surgery.

## Discussion

PEX is a common age-related disorder of the extracellular matrix and is frequently associated with severe chronic secondary open angle glaucoma and cataracts [2]. Recently, a mutation of the *LOXL1* gene was shown to be responsible for PEX, indicating a systemic abnormality in this clinical entity [3]. Evidence has been accumulating documenting the morphological alterations in almost all cell layers of the cornea in PEX [4]. The PEX can lead to corneal endothelial cell decompensation, which can result in severe BK, a vision-threatening disorder [2,5].

In our two cases, PEX was the only recognizable factor that could be responsible for the development of BK. To date, there has been no report on simultaneous description of the IVCM and electron microscopic findings in the PEX related BK in the literature. The IVCM findings in this report were in agreement with our earlier observations that the endothelial cell density was significantly decreased and PEX materials were precipitated on the endothelial cells. More interestingly, the PEX materials were also found in the stroma as detected by our immunohistochemical study.

The origin of the PEX materials is still controversial although fibroblastic changes of endothelial cells have been suggested to be responsible for PEX material formation [2]. Our findings also showed that IVCM is a useful noninvasive and rapid method for diagnosing PEX endotheliopathy. Early diagnosis of these patients before intraocular surgery or laser iridotomy is critical for better preservation of the endothelial function. Our recent survey showed that the PEX endotheliopathy comprises up to 50% of the unknown BK cases in Japan (Japanese Ministry of Health PEX Endotheliopathy Study Group). Alert should be raised to this unique clinical entity that relates to aging process, bilaterally involved and is probably more prevalent than we have believed.

## Conclusions

Our in vivo confocal microscopic and histopathological studies showed the endothelial dysfunction of the two cases are probably associated with pseudoexfoliation syndrome. PEX related corneal endotheliopathy should be taken into consideration as one possible etiology for bullous keratopathy of unknown origin. In vivo confocal microscopy is a useful tool for assistance of the diagnosis.

### Consent

Written informed consent was obtained from the two patients for publication of this report and any accompanying images.

## Competing interests

The authors declare that they have no competing interests.

## Authors’ contributions

XZ, YI and YH performed the examination and operation of the two cases. AS, TG and YO conceived of the design of this report. All authors read and approved the final manuscript.

## Funding

The authors have no proprietary interest in any materials or methods described within this article.

“Supported by a grant from the Ministry of Health, Labor and Welfare, Japan”.

## Pre-publication history

The pre-publication history for this paper can be accessed here:

http://www.biomedcentral.com/1471-2415/12/17/prepub
